# Mass Transpiration in Nonlinear MHD Flow Due to Porous Stretching Sheet

**DOI:** 10.1038/s41598-019-52597-5

**Published:** 2019-12-06

**Authors:** Jitender Singh, U. S. Mahabaleshwar, Gabriella Bognár

**Affiliations:** 1 Department of Mathematics, Guru Nanak Dev University, Amritsar, 143005 India; 2 Department of Mathematics, Davangere University Shivagangotri, Davangere, 577 007 India; 3 Institute of Machine and Product Design, University of Miskolc, Miskolc-Egyetemvaros, 3515 Hungary

**Keywords:** Mathematics and computing, Applied mathematics

## Abstract

Motivated from numerous practical applications, the present theoretical and numerical work investigates the nonlinear magnetohydrodynamic (MHD) laminar boundary layer flow of an incompressible, viscous fluid over a porous stretching sheet in the presence of suction/injection (mass transpiration). The flow characteristics are obtained by solving the underlying highly nonlinear ordinary differential equation using homotopy analysis method. The effect of parameters corresponding to suction/injection (mass transpiration), applied magnetic field, and porous stretching sheet parameters on the nonlinear flow is investigated. The asymptotic limits of the parameters regarding the flow characteristics are obtained mathematically, which compare very well with those obtained using the homotopy analysis technique. A detailed numerical study of the laminar boundary layer flow in the vicinity of the porous stretching sheet in MHD and offers a particular choice of the parametric values to be taken in order to practically model a particular type of the event among suction and injection at the sheet surface.

## Introduction

The steady, laminar MHD boundary layer flows driven by moving boundaries are among the classical problems of theoretical fluid mechanics (see Schlichting^1^). The usual stretching sheet problems arise in polymer extrusion processes that involve the cooling of continuous strips extruded from a dye by drawing them horizontally through a stagnant cooling fluid^2^.

The phenomena of momentum transfer in steady, laminar boundary layer flows have received much attention due to their wide applications which include drawing of plastics and elastic sheets, metal and polymer expulsion forms, paper creation, and cooling of metallic sheets etc.^2,3^.

Viscous fluid flow past a linear stretching sheet is a classical problem of laminar boundary layer flow. Blasius^4^ first discovered the boundary layer flow on a flat plate using similarity transformations. Sakiadis^5,6^ investigated the steady laminar boundary layer flow on a moving plate in a quiescent liquid and obtained both closed form as well as approximate solutions. Crane^7^ considered the flow due to stretching of plastic sheets in the polymer industry and obtained an analytical solution of the laminar boundary layer equations. Recently, Al-Housseiny and Stone^8^ have investigated the laminar boundary layer flow due to motion of stretching sheets by taking into account both the fluid motion as well as the motion of the sheet.

The control of the boundary layer flow due to stretching sheet can be enhanced by introducing magnetohydrodynamic (MHD) effects. This can be done by taking an electrically conducting fluid above the sheet and applying a magnetic field perpendicular to the plane of the sheet. In this connection, Pavlov^9^ was the first to investigate the MHD flow past a stretching sheet using the Hartman formulation. He found that the applied magnetic field and permeability cause depletion of the boundary layer thickness near the sheet surface. Chakrabarti and Gupta^10^ extended the classical work of Crane^7^ to include the effect of a transverse magnetic field and obtained the analytical solutions to the MHD flow over a stretching sheet.

Siddheshwar and Mahabaleshwar^3^ investigated MHD viscoelastic fluid flow and heat transfer over a stretching sheet in the presence of radiations using Chandrasekhar formulation^11^. Thereafter, many authors have investigated the MHD boundary layer flows past a stretching/shrinking sheet with different control parameters and conditions^12–23^.

Boundary layer flows through saturated porous media and MHD has gained significant attention in the recent times because of their promising engineering applications, such as in moisture transport in thermal insulation, ceramic processing, extraction of geothermal energy, nuclear reactor cooling systems, underground nuclear waste disposal, ground water pollution control, and filtration processes.

The objective of the present study is to investigate the boundary layer fluid flow over a permeable stretching sheet. The fluid under consideration is taken as electrically conducting and subjected to an externally imposed magnetic field normal to the stretching surface. The flow, mass transfer, and MHD effects are examined by applying the well known homotopy analysis method^1^^1^ ^24^ (hereafter referred to as HAM)^13,25,26^ to the highly nonlinear ordinary differential equations governing the flow. The HAM was originally proposed and developed by Liao^24,25,27–31^. This method is suitable for investigations of the present type as is evident from its use in the earlier studies^13^ regarding ease of its applicability and accuracy for obtaining the solution of nonlinear ordinary differential equations. There is another powerful variant of HAM known as optimal homotopy perturbation method (OHPM)^32,33^ available in literature to obtain analytic solutions of nonlinear differential equations. However, we will not use OHPM in the present work since HAM is sufficient for our purpose. The present study focuses on obtaining nonlinear numerical calculations relevant for visualizing the changes happening in the boundary layer flow generated due to stretching of the sheet by application of the external magnetic field, the porosity of the medium around the sheet, and suction/injection of the fluid at the sheet surface.

## Mathematical Model

**Figure 1:**
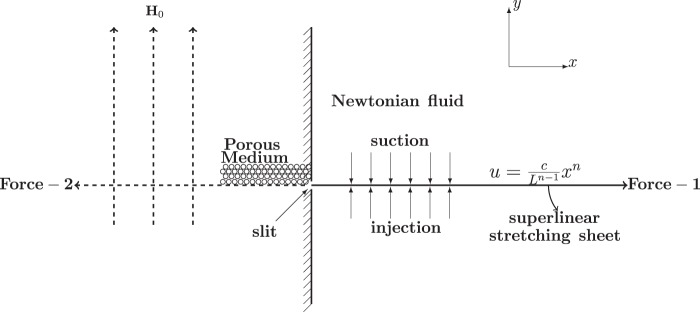
Diagram showing flow of an electrically conducting, incompressible Newtonian fluid through a porous medium over a stretching sheet issuing from the slit at the origin of the rectangular coordinates (*x*, *y*) with suction/injection. The system is subjected to a vertical magnetic field **H**_0_.

We consider the steady two dimensional flow of an electrically conducting, incompressible Newtonian fluid through a porous medium over a stretching sheet issuing from a slit at the origin of the rectangular coordinates as shown in Fig. 1. The sheet is assumed to be horizontal and coincides with the plane *y* = 0. Two equal and opposite forces are applied to the sheet to stretch it along the *x*-axis. The velocity of the stretching is (*U*(*x*),0), where

1 $$U(x)={U}_{0}{(x/L)}^{n}$$

such that *n* > −1 is the stretching parameter; *U*_0_ and *L* are the characteristic scales for measuring the velocity of the stretching and distance, respectively. The porous medium is assumed to have permeability

2 $$\kappa (x)={\kappa }_{0}{(x/L)}^{1-n}$$

and is subjected to an external vertical magnetic field

3 $${{\bf{H}}}_{0}(x)=(0,{H}_{0}{(x/L)}^{(n-1)/2}),$$

where *κ*_0_ and *H*_0_ denote the characteristic scales for measuring the permeability of the porous medium and magnetic field, respectively. The electrical conductivity *σ* of the fluid is assumed to be small so that the induced magnetic field in the fluid is weak as compared to the applied magnetic field. The sheet is permeable and subjected to suction velocity (0,*V*(*x*)) (see [^1^, Ch. 11, pp. 302]), where

4 $$V(x)=-{V}_{0}\sqrt{\frac{n+1}{2}}{(x/L)}^{(n-\mathrm{1)/2}};\,n > -1.$$

As a convention, *V*(*x*) > 0 implies suction while *V*(*x*) < 0 implies injection of the fluid at *y* = 0.

The stretching of the sheet induces a fluid velocity field (*u*(*x*, *y*), *v*(*x*, *y*)) which satisfies the equation of continuity

5 $$\frac{\partial u}{\partial x}+\frac{\partial v}{\partial y}=0$$

and the boundary layer approximation of the momentum equation

6 $$u\frac{\partial u}{\partial x}+v\frac{\partial u}{\partial y}=\nu \frac{{\partial }^{2}u}{\partial {y}^{2}}-[\frac{\sigma H{(x)}^{2}}{\rho }+\frac{\nu }{\kappa (x)}]u,$$

where *ν* is the kinematic viscosity and *ρ* is the fluid density. The boundary conditions for the flow are given by

7 $$u(x,0)=U(x);\,v(x,0)=V(x);\,\mathop{\mathrm{lim}}\limits_{y\to \infty }u(x,y)=0,$$

where *U*(*x*) and *V*(*x*) are as in (1) and (4).

We use similarity transformations to convert the system (5) and (6) in simpler form. Let *ψ* be the stream function for the flow, such that $u=\frac{\partial \psi }{\partial y}$ and $v=-\,\frac{\partial \psi }{\partial x}$. Introducing the Reynolds number ${\mathcal R} e$ and the similarity variable *η*, such that

8 $$ {\mathcal R} e={U}_{0}L/\nu  > 0;\,\eta =\sqrt{ {\mathcal R} e(n+1)/2}{(x/L)}^{\frac{n-1}{2}}y/L > 0,$$

and

9 $$\psi =\nu \sqrt{2 {\mathcal R} e/(n+1)}{(x/L)}^{\frac{n+1}{2}}f(\eta ),$$

where *f*(*η*) denotes the dimensionless form of the stream function, we have

10 $$u=U(x)f^{\prime} (\eta );\,v=(V(x)/{V}_{c})[f(\eta )+\frac{n-1}{n+1}\eta \,f^{\prime} (\eta )],$$

where

11 $${V}_{c}=({V}_{0}L/\nu )/\sqrt{ {\mathcal R} e}$$

is the dimensionless measure of suction/injection known as the mass transpiration parameter^22^.

Using (9) and (10) in (5) and (6), we have the following nonlinear third order system

12 $$\begin{array}{c}{f}^{^{\prime\prime\prime} }+f{f}^{{\rm{^{\prime} }}{\rm{^{\prime} }}}-\frac{2n}{n+1}{f}^{{}^{{\rm{^{\prime} }}}2}-\frac{2 {\mathcal M} }{n+1}{f}^{{\rm{^{\prime} }}}=0;\,f(0)={V}_{c};\,{f}^{{\rm{^{\prime} }}}\,(0)=1;\,\mathop{{\rm{l}}{\rm{i}}{\rm{m}}}\limits_{\eta \to {\rm{\infty }}}{f}^{{\rm{^{\prime} }}}\,(\eta )=0,\end{array}$$

where 0 ≤ *η* < ∞. The stretching parameter *n*, the mag*n*etic parameter ${\mathcal M} ={\mathscr Q}+\kappa$ appear in (12), where

13 $${\mathscr Q}=\sigma {L}^{2}{H}_{0}^{2}/(\rho \nu  {\mathcal R} e);\,\kappa ={L}^{2}/({\kappa }_{0} {\mathcal R} e\mathrm{)}.$$

The symbol $\kappa$ is the dimensionless form of the porosity of the medium so that ${\kappa }^{-1}$ is the dimensionless measure of permeability. Also, the parameter ${\mathscr Q}$ is related to the Chandrasekhar number *Q* by ${\mathscr Q}=Q/ {\mathcal R} e$, where *Q* = *σL*^2^*H*_0_^2^/(*ρν*). Observe that *Q* ≥ 0 and $\kappa  > 0$. If no magnetic field is applied, ${\mathscr Q}=0$ so that ${\mathcal M} =\kappa$. Increasing the permeability of the porous medium lowers the value of $\kappa$ and hence of ${\mathcal M}$. If the me0dium is not porous, the term containing porosity is absent which correspond to $\kappa \to 0$ and ${\mathcal M} ={\mathscr Q}$. On the other hand, if the medium is not porous and no magnetic field is applied then ${\mathcal M} \to 0$. Also note that *V*_*c*_ < 0 for suction and *V*_*c*_ > 0 for injection. Finally, recall that *n* > −1.

## Solution

To solve the nonlinear system (12), we use the well known HAM^25,31^ which is described as follows. For *p* ∈ [0,1] as the homotopy embedding parameter, we consider the following boundary value problem

14 $$\begin{array}{c}{f}^{{\rm{^{\prime} }}{\rm{^{\prime} }}{\rm{^{\prime} }}}-{\alpha }^{2}{f}^{{\rm{^{\prime} }}}+p\{f{f}^{{\rm{^{\prime} }}{\rm{^{\prime} }}}-\frac{2n}{n+1}{f}^{{}^{{\rm{^{\prime} }}}2}+({\alpha }^{2}-\frac{2 {\mathcal M} }{n+1})\,{f}^{{\rm{^{\prime} }}}\}=0,\,\\ f(0)={V}_{c},\,\\ {f}^{{\rm{^{\prime} }}}(0)=1,\,\\ \mathop{{\rm{l}}{\rm{i}}{\rm{m}}}\limits_{\eta \to {\rm{\infty }}}{f}^{{\rm{^{\prime} }}}(\eta )=0.\end{array}$$

where *α* ≠ 0 is the unknown scalar to be determined. For *p* = 0, the system (14) gives the linear system *f* ′′′−*α*^2^*f* ′ = 0 and for *p* = 1, it is the nonlinear system (12). Now assume a solution of (14) in the form

$$f={f}_{0}+p{f}_{1}+{p}^{2}{f}_{2}+\cdots +{p}^{k}{f}_{k}+\cdots $$

and compare the like powers of *p* to obtain the following sequence of boundary value problems

15a $${{f}^{{\rm{^{\prime} }}{\rm{^{\prime} }}{\rm{^{\prime} }}}}_{0}-{\alpha }^{2}{{f}^{{\rm{^{\prime} }}}}_{0}=0;\,{f}_{0}(0)={V}_{c};\,{{f}^{{\rm{^{\prime} }}}}_{0}(0)=1;\,\mathop{{\rm{l}}{\rm{i}}{\rm{m}}}\limits_{\eta \to {\rm{\infty }}}{{f}^{{\rm{^{\prime} }}}}_{0}(\eta )=0,$$

15b $$\begin{array}{c}{{f}^{{\rm{^{\prime} }}{\rm{^{\prime} }}{\rm{^{\prime} }}}}_{k}-{\alpha }^{2}{{f}^{{\rm{^{\prime} }}}}_{k}+({\alpha }^{2}-\frac{2 {\mathcal M} }{n+1}){{f}^{{\rm{^{\prime} }}}}_{k-1}+\mathop{\sum }\limits_{m=0}^{k-1}\,({f}_{m}{{f}^{{\rm{^{\prime} }}{\rm{^{\prime} }}}}_{k-1-m}-\frac{2n}{n+1}{{f}^{{\rm{^{\prime} }}}}_{m}{{f}^{{\rm{^{\prime} }}}}_{k-1-m})=0,\\ {f}_{k}(0)={{f}^{{\rm{^{\prime} }}}}_{k}(0)=\mathop{{\rm{l}}{\rm{i}}{\rm{m}}}\limits_{\eta \to {\rm{\infty }}}{{f}^{{\rm{^{\prime} }}}}_{k}(\eta )=0,\,k=1,\,2,\,\ldots .\end{array}$$

The system (15) and (16) can be solved recursively starting with *k* = 0, where at each later stage, one needs to solve a linear boundary value problem using the solutions obtained from all of the previous stages. We have obtained the solutions of first four of these problems in closed form as follows.

16a $${f}_{0}(\eta )={V}_{c}+\frac{1-\exp \{\,-\,\alpha \eta \}}{\alpha },$$

16b $$\begin{array}{ccc}{f}_{1}(\eta ) & = & \frac{1}{6{\alpha }^{3}}(3\alpha (\alpha -{V}_{c})-2-\beta -3 {\mathcal M} (2-\beta ))\\  &  & +\,\frac{1}{6{\alpha }^{3}}(1+2\beta +3 {\mathcal M} (2-\beta )+3\alpha ({V}_{c}-\alpha ))\exp \{-\alpha \eta \}\\  &  & +\,\frac{1}{2{\alpha }^{2}}(1+ {\mathcal M} (2-\beta )+\alpha ({V}_{c}-\alpha ))\,\eta \,\exp \{-\alpha \eta \}+\frac{(1-\beta )}{6{\alpha }^{3}}\exp \{-2\alpha \eta \},\end{array}$$

16c $$\begin{array}{rcl}{f}_{2}(\eta ) & = & \frac{{q}_{0}}{72{\alpha }^{5}}-\frac{1}{144{\alpha }^{5}}({q}_{1}+6{q}_{2}\alpha \eta +6{q}_{3}{\alpha }^{2}{\eta }^{2})\\  &  & \times \exp \{\,-\,\alpha \eta \}-\frac{\mathrm{(1}-\beta )}{36{\alpha }^{5}}({q}_{4}+{q}_{5}\alpha \eta )\exp \{\,-\,2\alpha \eta \}\\  &  & +\,\frac{\mathrm{(1}-\beta \mathrm{)(4}\beta -\mathrm{5)}}{144{\alpha }^{5}}\exp \{\,-\,3\alpha \eta \},\end{array}$$

16d $$\begin{array}{rcl}{f}_{3}(\eta ) & = & \frac{{r}_{0}}{4320{\alpha }^{7}}+\frac{1}{8640{\alpha }^{7}}({r}_{1}+30{r}_{2}\alpha \eta +360{r}_{3}{\alpha }^{2}{\eta }^{2}+180{r}_{4}{\alpha }^{3}{\eta }^{3})\\  &  & \times \exp \{\,-\,\alpha \eta \}-\frac{\mathrm{(1}-\beta )}{432{\alpha }^{7}}({r}_{5}+{r}_{6}\alpha \eta +{r}_{7}{\alpha }^{2}{\eta }^{2})\exp \{\,-\,2\alpha \eta \}\\  &  & +\,\frac{\mathrm{(1}-\beta \mathrm{)(5}-4\beta )}{1728{\alpha }^{7}}({r}_{8}+18{r}_{9}\alpha \eta )\exp \{\,-\,3\alpha \eta \}\\  &  & +\,\frac{\mathrm{(1}-\beta \mathrm{)(33}-51\beta +20{\beta }^{2})}{4320{\alpha }^{7}}\exp \{\,-\,4\alpha \eta \},\end{array}$$

where

17 $$\beta =\frac{2n}{1+n},$$

18 $$\begin{array}{ccc}{q}_{0} & = & 10+2\beta (2\beta +9 {\mathcal M} (2-\beta ))+13\beta \\  &  & +\,9 {\mathcal M} (2-\beta )(4+3 {\mathcal M} (2-\beta ))+2(11+7\beta +18 {\mathcal M} (2-\beta )){V}_{c}\alpha \\  &  & -\,9(4+2\beta +6 {\mathcal M} (2-\beta )-{V}_{c}^{2}){\alpha }^{2}-36{\alpha }^{3}+27{\alpha }^{4},\end{array}$$

19 $$\begin{array}{ccc}{q}_{1} & = & 3+4\beta (5\beta +18 {\mathcal M} (2-\beta ))+31\beta \\  &  & +\,18 {\mathcal M} (2-\beta )(2+3 {\mathcal M} (2-\beta ))+8(2+7\beta +9 {\mathcal M} (2-\beta )){V}_{c}\alpha \\  &  & -\,18(2+4\beta +6 {\mathcal M} (2-\beta )-{V}_{c}^{2}){\alpha }^{2}-72{\alpha }^{3}+54{\alpha }^{4},\end{array}$$

20 $$\begin{array}{ccc}{q}_{2} & = & 3+2\beta (3+2 {\mathcal M} (2-\beta ))\\  &  & +\,14 {\mathcal M} (2-\beta )(1+9 {\mathcal M} (2-\beta ))+4(2+\beta +3 {\mathcal M} (2-\beta )){V}_{c}\alpha \\  &  & -\,(2(7+2\beta +9 {\mathcal M} (2-\beta ))-3{V}_{c}^{2}){\alpha }^{2}-12{\alpha }^{3}+9{\alpha }^{4},\end{array}$$

21 $$\begin{array}{c}{q}_{3}=3(1+ {\mathcal M} (2-\beta {))}^{2}+2(1+ {\mathcal M} (2-\beta )){V}_{c}\alpha \\ \,\,\,\,\,-\,3(2(1+ {\mathcal M} (2-\beta ))-{V}_{c}^{2}){\alpha }^{2}-6{\alpha }^{3}+3{\alpha }^{4},\end{array}$$

22 $${q}_{4}=3+4\beta +9 {\mathcal M} (2-\beta )+7{V}_{c}\alpha -9{\alpha }^{2};\,{q}_{5}=6(1+ {\mathcal M} (2-\beta )+{V}_{c}\alpha -{\alpha }^{2}),$$

23 $$\begin{array}{ccc}{r}_{0} & = & -\,226-\beta (587+437\beta +100{\beta }^{2})-150 {\mathcal M} \{(2+\beta )(5+4\beta +9 {\mathcal M} )+9{ {\mathcal M} }^{2}\}\\  &  & -\,5(125+223\beta +84{\beta }^{2}+528 {\mathcal M} +336\beta  {\mathcal M} +432{ {\mathcal M} }^{2}){V}_{c}\alpha \\  &  & +\,10(150+195\beta +60{\beta }^{2}+540 {\mathcal M} +270\beta  {\mathcal M} +405{ {\mathcal M} }^{2}-40{V}_{c}^{2}-41\beta {V}_{c}^{2}-81 {\mathcal M} {V}_{c}^{2}){\alpha }^{2}\\  &  & +\,240(11+7\beta +18 {\mathcal M} ){V}_{c}{\alpha }^{3}-270(10+5\beta +15 {\mathcal M} -3{V}_{c}^{2}){\alpha }^{4}-2160{V}_{c}{\alpha }^{5}+1350{\alpha }^{6},\end{array}$$

24 $$\begin{array}{ccc}{r}_{1} & = & -\,9+677\beta +1472{\beta }^{2}+560{\beta }^{3}+150 {\mathcal M} (3+31\beta +20{\beta }^{2}+18 {\mathcal M} +36\beta  {\mathcal M} +18{ {\mathcal M} }^{2})\\  &  & +\,5\{(-9+445\beta +428{\beta }^{2}+384 {\mathcal M} +1344\beta  {\mathcal M} +864{ {\mathcal M} }^{2}){V}_{c}\}\alpha \\  &  & +\,10\{-15(3+31\beta +20{\beta }^{2})-270 {\mathcal M} (2+4\beta +3 {\mathcal M} )+2(-1+82\beta +81 {\mathcal M} ){V}_{c}^{2}\}{\alpha }^{2}\\  &  & -\,960(2+7\beta +9 {\mathcal M} ){V}_{c}{\alpha }^{3}-540\{-5(1+2\beta +3 {\mathcal M} )+3{V}_{c}^{2}\}{\alpha }^{4}+4320{V}_{c}{\alpha }^{5}-2700{\alpha }^{6},\end{array}$$

25 $$\begin{array}{ccc}{r}_{2} & = & 1+53\beta +36{\beta }^{2}+5 {\mathcal M} (15+35\beta +4{\beta }^{2})+6{ {\mathcal M} }^{2}(31+14\beta +15 {\mathcal M} )\\  &  & +\,\{9+115\beta +20{\beta }^{2}+16 {\mathcal M} (10+8\beta +9 {\mathcal M} )\}{V}_{c}\alpha \\  &  & -\,\{75+175\beta +20{\beta }^{2}+6 {\mathcal M} (62+28\beta +45 {\mathcal M} )-2(5+22\beta +27 {\mathcal M} ){V}_{c}^{2}\}{\alpha }^{2}\\  &  & -\,32(5+4\beta +9 {\mathcal M} ){V}_{c}{\alpha }^{3}+6(31+14\beta +45 {\mathcal M} -9{V}_{c}^{2}){\alpha }^{4}+144{V}_{c}{\alpha }^{5}-90{\alpha }^{6},\end{array}$$

26 $$\begin{array}{ccc}{r}_{3} & = & (1+\beta )(1+2\beta +5 {\mathcal M} +\beta  {\mathcal M} +3{\beta }^{2})+(3+3\beta +10 {\mathcal M} +2\beta  {\mathcal M} +6{ {\mathcal M} }^{2}){V}_{c}\alpha \\  &  & -\,(6+3\beta + {\mathcal M} (16+2\beta +9 {\mathcal M} )-(2+\beta +3 {\mathcal M} ){V}_{c}^{2}){\alpha }^{2}-2(5+\beta +6 {\mathcal M} ){V}_{c}{\alpha }^{3}\\  &  & +\,(8+\beta +9 {\mathcal M} -3{V}_{c}^{2}){\alpha }^{4}+6{V}_{c}{\alpha }^{5}-3{\alpha }^{6},\end{array}$$

27 $$\begin{array}{ccc}{r}_{4} & = & {(1+ {\mathcal M} )}^{3}+3(1+ {\mathcal M} {)}^{2}{V}_{c}\alpha -3(1+ {\mathcal M} )(1+ {\mathcal M} -{V}_{c}^{2}){\alpha }^{2}\\  &  & -\,\{6(1+ {\mathcal M} )-{V}_{c}^{2}\}{V}_{c}{\alpha }^{3}+\,3(1+ {\mathcal M} -{V}_{c}^{2}){\alpha }^{4}+3{V}_{c}{\alpha }^{5}-{\alpha }^{6},\end{array}$$

28 $$\begin{array}{ccc}{r}_{5} & = & 6+49\beta +28{\beta }^{2}+15 {\mathcal M} (6+8\beta +9 {\mathcal M} )\\  &  & +\,4(9+22\beta +42 {\mathcal M} ){V}_{c}\alpha -(90+120\beta +270 {\mathcal M} -41{V}_{c}^{2}){\alpha }^{2}-\,168{V}_{c}{\alpha }^{3}+135{\alpha }^{4},\\  & \end{array}$$

29 $$\begin{array}{ccc}{r}_{6} & = & 2(21+30\beta +90 {\mathcal M} +24\beta  {\mathcal M} +63{ {\mathcal M} }^{2})+(120+48\beta +192 {\mathcal M} ){V}_{c}\alpha \\  &  & -\,6(30+8\beta +42 {\mathcal M} -11{V}_{c}^{2}){\alpha }^{2}-192{V}_{c}{\alpha }^{3}+126{\alpha }^{4},\end{array}$$

30 $${r}_{7}=36+72 {\mathcal M} +36{ {\mathcal M} }^{2}+72(1+ {\mathcal M} ){V}_{c}\alpha -36(2(1+ {\mathcal M} )-{V}_{c}^{2}){\alpha }^{2}-72{V}_{c}{\alpha }^{3}+36{\alpha }^{4},$$

31 $${r}_{8}=11+12\beta +30 {\mathcal M} +23{V}_{c}\alpha -30{\alpha }^{2};{r}_{9}=1+ {\mathcal M} +\alpha ({V}_{c}-\alpha ).$$

The expressions for the functions *f*_*k*_ for *k* > 3 are lengthy; so, we omit them. The order of approximation of *f* using HAM is the integer *N* such that $f\approx {\sum }_{k=0}^{N}\,{f}_{k}$. In the present work, we have obtained up to seventh order approximation of *f*. All numerical computations have been done using computer programming in MATLAB.

The HAM allows us to choose the scalar *α* appropriately. We take

32 $$\alpha =-\,f^{\prime\prime} \mathrm{(0)}\approx -\mathop{\sum }\limits_{m\mathrm{=0}}^{N}\,{f^{\prime\prime} }_{m}\mathrm{(0)}=\alpha -\mathop{\sum }\limits_{m\mathrm{=1}}^{N}\,{f^{\prime\prime} }_{m}\mathrm{(0),}$$

where we have *f'*_0_(0) = −*α* from (16a). From (32), we have ${\sum }_{m=1}^{N}\,{f^{\prime\prime} }_{m}(0)\approx 0$, which can be solved numerically to obtain an approximate value of *α*, depending upon *N* and the dimensionless parameters. For *N* = 1, (32) gives the following closed form expression

33 $$\alpha \approx \frac{{V}_{c}}{2}+\frac{1}{2}\sqrt{{V}_{c}^{2}+\frac{4}{3}\{1+2\beta +3 {\mathcal M} (2-\beta )\}},$$

which is valid for all *n* ≥ 0 as ${V}_{c}^{2}+\frac{4}{3}\{1+2\beta +3 {\mathcal M} (2-\beta )\}\ge 0$ holds since ${\mathcal M}  > 0$ and 0 ≤ *β* < 2 for all *n* ≥ 0. For −1 < *n* < 0, *β* ∈ (−∞,0). In this case, (33) holds for ${\mathcal M} =2/3$. For ${\mathcal M} \ne 2/3$, (33) holds either for all *β* ∈ (−∞,0) and ${\mathcal M}  > 2/3$ or for *β* ≥ *β*_0_ and ${\mathcal M}  < 2/3$, where

34 $${\beta }_{0}=-\frac{3{V}_{c}^{2}+4+24 {\mathcal M} }{4(2-3 {\mathcal M} )}.$$

The present formula (33) recovers the value of *f* ′′(0) as in Crane^7^ on taking ${V}_{c}\to 0= {\mathcal M}$ and *β* = 1, Pavlov^9^ on taking *V*_*c*_→0 and *β* = 1, Gupta and Gupta^34^ for *β* = 1, and Hayat *et al*.^14^ and Rashidi^15^ for *V*_*c*_→0.

**Figure 2:**
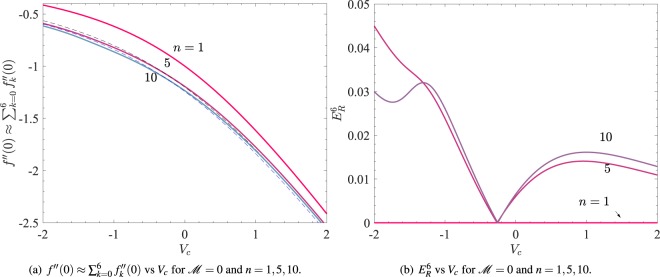
The solid thick curves and the thin dashed curves have been obtained for *N* = 6 and *N* = 1, respectively.

To compare the first order approximation of *f* ′′(0) by (33) with the higher order approximations, we have obtained Fig. 2(a) which shows the variation of $f^{\prime\prime} \mathrm{(0)}\approx {\sum }_{k\mathrm{=0}}^{N}\,{f^{\prime\prime} }_{k}\mathrm{(0)}$ with *V*_*c*_ for ${\mathcal M} =0$ and *n* = 1, 5, 10. The solid thick curves and the thin dashed curves have been obtained for *N* = 6 and *N* = 1, respectively. Clearly for each *n*, the formula (33) gives good approximation to *f* ′′(0). To quantify this approximation, we define

35 $${E}_{R}^{N}=|\mathop{\sum }\limits_{k=0}^{N}\,{{f}^{{\rm{^{\prime} }}{\rm{^{\prime} }}}}_{k}(0)+\frac{{V}_{c}}{2}+\frac{1}{2}\sqrt{{V}_{c}^{2}+\frac{4}{3}\{1+2\beta +3 {\mathcal M} (2-\beta )\}}|/|\mathop{\sum }\limits_{k=0}^{N}\,{{f}^{{\rm{^{\prime} }}{\rm{^{\prime} }}}}_{k}(0)|,$$

which is the relative difference in the values of *f* ′′(0) = −*α* obtained using first order approximation with respect to the *N*-th order approximation. Figure 2(b) shows the variation of *E*_*R*_^6^ with *V*_*c*_ for the same parametric values as in Fig. 2(a). It is clear from the Fig. 2(a) that the relative difference *E*_*R*_^6^ = 0 for *n* = 1, and it remains less than 5% for the other two values of *n*.

Table 1 shows the numerical value of *α* obtained with the higher order approximations for *V*_*c*_ → 0, ${\mathcal M} =0$, and *n* = 10. Clearly the method converges for *N* = 3 within the tolerance of 10^−2^. For *N* = 7 and *V*_*c*_ → 0, we have *f* ′′(0) ≈ −1.2349677, which is close to the corresponding numerical value −1.234875 obtained by Vajravelu and Cannon^35^ and Cortell^36^.

**Table 1:** Dependence of *α* on *N* and *V*_*c*_ for ${\mathcal M} =0$ and *n* = 10.

	*N*→	1	2	3	4	5	6	7
*V*_*c*_								
2	*α*	2.5954481	2.5786412	2.5699411	2.5656288	2.5635278	2.5624998	2.5619887
1	*α*	1.8399457	1.8231932	1.8158511	1.8127370	1.8113701	1.8107333	1.8104193
0	*α*	1.2431631	1.2371618	1.2358824	1.2353717	1.2351441	1.2350297	1.2349677
−1	*α*	0.8399457	0.8947983	0.8499919	0.8673482	0.8628021	0.8624300	0.8639661
−2	*α*	0.5954481	0.5205083	0.5457379	0.5283839	0.5349155	0.5315083	0.5330783

**Figure 3:**
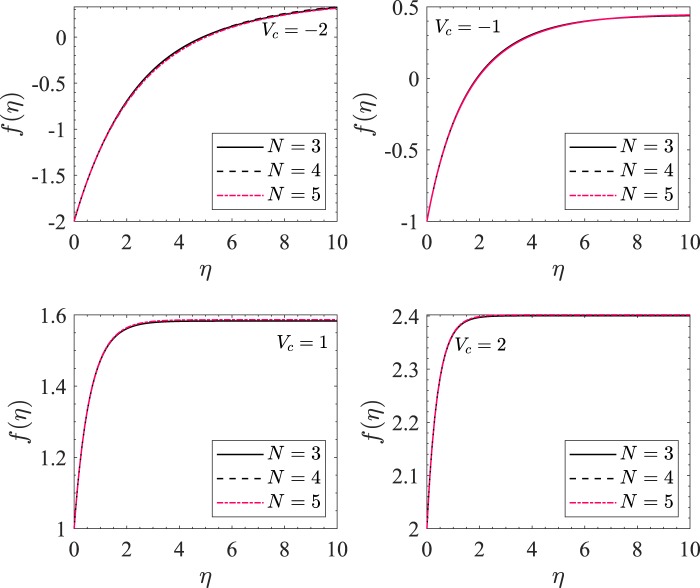
*f*(*η*) vs *η* for *n* = 10 and ${\mathcal M} =0$. The different subfigures are obtained for different values of *V*_*c*_.

To compare the third order approximation (*N* = 3) of HAM to the solution with the higher order approximations, we have plotted *f*(*η*) with respect to *η* in Fig. 3 for *n* = 10 and ${\mathcal M} =0$. We have taken ${\mathcal M} =0$ since the convergence of the method is comparatively rapid for  ${\mathcal M}$ > 0. The other parametric values are chosen to test the extreme case where the error can possibly be maximum. The three curves in each subfigure correspond to *N* = 3, 4, and 5, respectively as shown in the legend. Clearly, the three curves are indistinguishable for each value of *V*_*c*_ which shows that the method converges for *N* = 3 and justifies that the third order analytic approximation to *f* using HAM are sufficient to describe the solution correctly. For rest of the numerical calculations, we have taken 4 ≤ *N* ≤ 7.

## Asymptotic Analysis

To understand the full parametric dependence of the present boundary layer flow, we obtain approximate analytic solution *f* for the following extreme cases.

**Case I**: $$ {\mathcal M} \gg 1$$

Let $\eta ={ {\mathcal M} }^{r}\hat{\eta }$ for some nonzero real number *r* when ${\mathcal M}$ is large, where $\hat{\eta }=O\mathrm{(1)}$. Assume

$$f={V}_{c}+{ {\mathcal M} }^{g}F(\hat{\eta })+O({ {\mathcal M} }^{2g}),$$

where *g* < 0. The boundary condition *f*(0) = *V*_*c*_ gives *F*(0) = 0, and *f* ′(0) = 1 implies ${ {\mathcal M} }^{g-r}{F}^{{\rm{^{\prime} }}}(0)=1$, so that *g* = *r* and *F*′(0) = 1. Now using (12), we have at the leading order ${ {\mathcal M} }^{2r}$

$$\begin{array}{c}{ {\mathcal M} }^{-2r-1}{F}^{{\rm{^{\prime} }}{\rm{^{\prime} }}{\rm{^{\prime} }}}+{V}_{c}{ {\mathcal M} }^{-r-1}{F}^{{\rm{^{\prime} }}{\rm{^{\prime} }}}+{ {\mathcal M} }^{-1}(F{F}^{{\rm{^{\prime} }}{\rm{^{\prime} }}}-\frac{2n}{n+1}{F}^{{}^{{\rm{^{\prime} }}}2})-\frac{2}{n+1}{F}^{{\rm{^{\prime} }}}=0,\\ F(0)=0,\,{F}^{{\rm{^{\prime} }}}(0)=1,\,\mathop{{\rm{l}}{\rm{i}}{\rm{m}}}\limits_{\eta \to {\rm{\infty }}}\,{F}^{{\rm{^{\prime} }}}(\eta )=0,\end{array}$$

where *r* < 0, which for sufficiently large ${\mathcal M}$ requires −1− 2*r* = 0 or *r* = −1/2. So, for ${\mathcal M} \to {\rm{\infty }}$, we get

36 $${F}^{{\rm{^{\prime} }}{\rm{^{\prime} }}{\rm{^{\prime} }}}-\frac{2}{n+1}{F}^{{\rm{^{\prime} }}}=0,\,F(0)=0,\,{F}^{{\rm{^{\prime} }}}(0)=1,\,\mathop{\mathrm{lim}}\limits_{\eta \to {\rm{\infty }}}{F}^{{\rm{^{\prime} }}}(\eta )=0.$$

The solution of (36) is given by $F=\sqrt{\frac{n+1}{2}}(1-\exp \{-\sqrt{\frac{2}{n+1}}\hat{\eta }\})$. Thus, we have the following asymptotic solution for large ${\mathcal M}$.

37 $$f={V}_{c}+\sqrt{\frac{n+1}{2 {\mathcal M} }}(1-\exp \{-\sqrt{\frac{2 {\mathcal M} }{n+1}}\eta \})+O({ {\mathcal M} }^{-1}),\, {\mathcal M} \gg 1.$$

**Figure 4:**
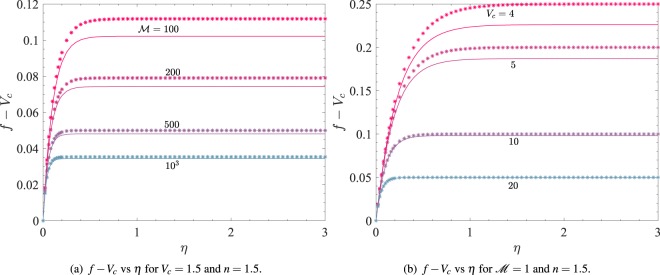
The points marked * correspond to (**a**) the asymptotic solution (37) and (**b**) the asymptotic solution (39). The other curves correspond to the numerical solution of (12).

The function *f* − *V*_*c*_ as obtained from (37) and (12) for large ${\mathcal M}$ is shown in Fig. 4(a) for *V*_*c*_ = 1.5 and *n* = 1.5. In each case, the points marked * correspond to the asymptotic solution (37), while the solid curves correspond to the numerical solution of (12). The asymptotic solution is in a maximum relative error of 0.6% for ${\mathcal M} ={10}^{2}$, which decreases rapidly on increasing ${\mathcal M}$. Thus the two solutions are in good agreement for ${\mathcal M} \ge {10}^{2}$.

**Case II**: *V*_*c*_ >> 1

For *V*_*c*_ >> 1, we take *η* = *V*_*c*_^*h*^η^*^, where ${\eta }^{\ast }=O\mathrm{(1)}$ and *h* is a nonzero scalar. Assume

$$f={V}_{c}+{V}_{c}^{q}G({\eta }^{\ast })+O({V}_{c}^{2q})$$

for large *V*_*c*_, where *q* < 0. Using the condition *f*(0) = *V*_*c*_ implies *G*(0) = 0 and *f*′(0) = 1 gives *G*′(0) = *V*_*c*_^*h*−*q*^, so that *G*′(0) = 1 and *h* = *q*. Now from (12), we have

$${G}^{{\rm{^{\prime} }}{\rm{^{\prime} }}{\rm{^{\prime} }}}+{V}_{c}^{1+q}{G}^{{\rm{^{\prime} }}{\rm{^{\prime} }}}-{V}_{c}^{2q}(\frac{2n}{n+1}{G}^{{}^{{\rm{^{\prime} }}}2}-\frac{2 {\mathcal M} }{n+1}{G}^{{\rm{^{\prime} }}})=0,\,G(0)=0,\,{G}^{{\rm{^{\prime} }}}(0)=1,\,\mathop{{\rm{l}}{\rm{i}}{\rm{m}}}\limits_{\eta \to {\rm{\infty }}}{G}^{{\rm{^{\prime} }}}(\eta )=0,$$

where *q* < 0, which for *V*_*c*_ >> 1 requires for a balance, 1 + *q* = 0 or *q* = −1. Thus, for *V*_*c*_ → ∞, we have

38 $${G}^{{\rm{^{\prime} }}{\rm{^{\prime} }}{\rm{^{\prime} }}}+{G}^{{\rm{^{\prime} }}{\rm{^{\prime} }}}=0,\,G(0)=0,\,{G}^{{\rm{^{\prime} }}}(0)=1,\,\mathop{{\rm{l}}{\rm{i}}{\rm{m}}}\limits_{\eta \to {\rm{\infty }}}{G}^{{\rm{^{\prime} }}}(\eta )=0,$$

which solves to *G* = 1 − *e*^−η*^ = 1 − *e*^−*V*^_*c*_^*η*^. Thus, in this case the asymptotic solution is given by

39 $$f={V}_{c}+\frac{1}{{V}_{c}}(1-\exp \{-{V}_{c}\eta \})+O({V}_{c}^{-2}),\,{V}_{c}\gg 1.$$

Figure 4b demonstrates a comparison between the asymptotic solution *f* − *V*_*c*_ as obtained from (39) marked * and the numerical solution of (12) (solid and dashed curves). The fixed parametric values are *n* = 1.5 and ${\mathcal M} =1$. Clearly, the two solutions are in very good agreement for *V*_*c*_ ≥ 4 within a relative error not exceeding 0.6%.

## Results and Discussion

The numerical results have been obtained for a wide range of parameters. For most of the numerical calculations, we have taken −2 ≤ *V*_*c*_ ≤ 2, 0 ≤ *n* ≤ 5, and $0 <  {\mathcal M} \le 100$. Even higher values of ${\mathcal M}$ are permissible since the method converges faster as ${\mathcal M}$ becomes larger, which can be seen from (33) through (16a)–(16d), since *α* = |*f* ′′(0)| which in turn rises with ${\mathcal M}$ for large ${\mathcal M}$. Also, we have taken at least the fourth order approximation of *f*, that is *N* ≥ 4 for the rest of the numerical calculations to meet the convergence issues using HAM. For obtaining the streamline patterns, we have taken 7th order approximation (*N* = 7) to *f*.

### Skin friction coefficient at sheet wall

If *τ* denotes the shear stress near the stretching sheet due to the fluid flow, we have

40 $$\tau =\mu \mathop{\mathrm{lim}}\limits_{y\to 0}(\frac{\partial u}{\partial y}+\frac{\partial v}{\partial x})=\mu (f^{\prime\prime} \mathrm{(0)}U\frac{\partial \eta }{\partial y}+\frac{dV}{dx}).$$

Then the coefficient *C*_*f*_ of the frictional drag which is also known as the skin friction parameter is defined for *x* > 0 as

41 $${C}_{f}=-\frac{\tau }{\frac{1}{2}\rho \{U{(x)}^{2}+V{(x)}^{2}\}}=\sqrt{\frac{\mathrm{2(}n+\mathrm{1)}}{ {\mathcal R} e}}\frac{\{-f^{\prime\prime} \mathrm{(0)}+\frac{(n-\mathrm{1)}{V}_{c}}{2 {\mathcal R} e}{(x/L)}^{-(n+\mathrm{1)/2}}\}}{\{{(x/L)}^{n+1}+\frac{(n+\mathrm{1)}{V}_{c}^{2}}{2 {\mathcal R} e}\}}.$$

Note that the skin friction coefficient *C*_*f*_ depends upon *f* ′′(0), *n*, ${\mathcal R} e$, *V*_*c*_, and the horizontal distance *x* from the slit, where *f* ′′(0) further depends upon ${\mathcal M}$, *n*, ${\mathcal R} e$, and *V*_*c*_.

In the literature, *f* ′′(0) is generally taken as a measure of the skin friction for a fixed *x*, which in the present situation holds only if there is no suction/injection, that is, *V*_*c*_ ≈ 0. If this is the case, we have

$$\mathop{\mathrm{lim}}\limits_{{V}_{c}\to 0}{C}_{f}=-\sqrt{\frac{\mathrm{2(}n+\mathrm{1)}}{ {\mathcal R} e}}{(x/L)}^{-(n+\mathrm{1)}}f^{\prime\prime} \mathrm{(0)}.$$

We first discuss the variation of *C*_*f*_ with *x* in the following three cases.

**Figure 5:**
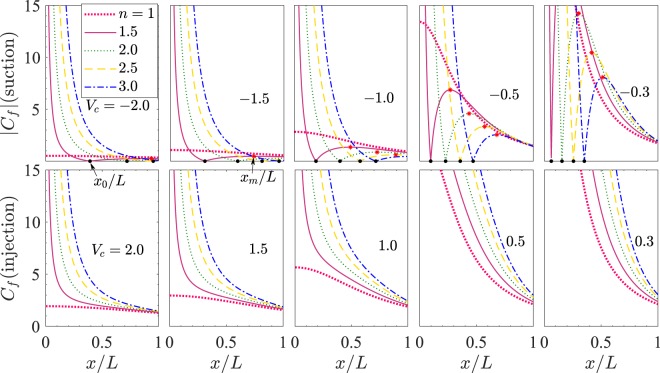
|*C*_*f*_| vs *x*/*L* for $$ {\mathcal M} =1$$ and $$ {\mathcal R} e=2$$. Each subfigure corresponds to one value of *V*_*c*_ among $$\mp 2.0$$, $$\mp 1.5$$, and $$\mp 1.0$$, $$\mp 0.5$$, and $$\mp 0.3$$. The different curves (labeled with different color and style as shown in the legend) in each subfigure correspond to different values of *n* ≥ 1 among 1, 1.5, 2.0, 2.5 and 3.0. Each black mark • corresponds to *x*_0_/*L* and each asterisk  shows the local maximum corresponding to *x* = *x*_*m*_.

**Case I**: *n* ≥ 1. Figure 5 depicts the variation of |*C*_*f*_| with *x*/*L* for ${\mathcal M} =1$ and ${\mathcal R} e=2$. Each subfigure corresponds to one value of *V*_*c*_ among $\mp 2.0$, $\mp 1.5$, $\mp 1.0$, $\mp 0.5$, and $\mp 0.3$. The different curves (labeled with different color and style as shown in the legend) in each subfigure correspond to different values of *n* ≥ 1 among 1, 1.5, 2.0, 2.5 and 3.0.

We first explain the curves for *V*_*c*_ = −2.0 which correspond to suction. We observe from (41) that *C*_*f*_ = 0 at *x* = *x*_0_ (see the black marks • in Fig. 5), where

42 $${x}_{0}/L={\{\frac{(n-\mathrm{1)}{V}_{c}}{2 {\mathcal R} ef^{\prime\prime} \mathrm{(0)}}\}}^{\mathrm{2/(}n+\mathrm{1)}}.$$

Clearly, *C*_*f*_ < 0 for *x* < *x*_0_ and *C*_*f*_ > 0 for *x* > *x*_0_. The skin friction coefficient *C*_*f*_ is negative for *x* in the interval (0,*x*_0_], where |*C*_*f*_| decreases rapidly from ∞ to 0. For *x* > *x*_0_, *C*_*f*_ is positive and starts increasing with further increase of *x* till a maximum is reached at about *x*/*L* = *x*_*m*_/*L* (see the points marked as  in Fig. 5), where (*x*_*m*_/*L*)^(*n*+1)/2^ is a real positive root of the following polynomial equation in *t*

43 $$8 {\mathcal R} {e}^{2}f^{\prime\prime} \mathrm{(0)}{t}^{3}-6 {\mathcal R} e(n-\mathrm{1)}{V}_{c}{t}^{2}-({n}^{2}-\mathrm{1)}{V}_{c}^{3}=0.$$

**Figure 6:**
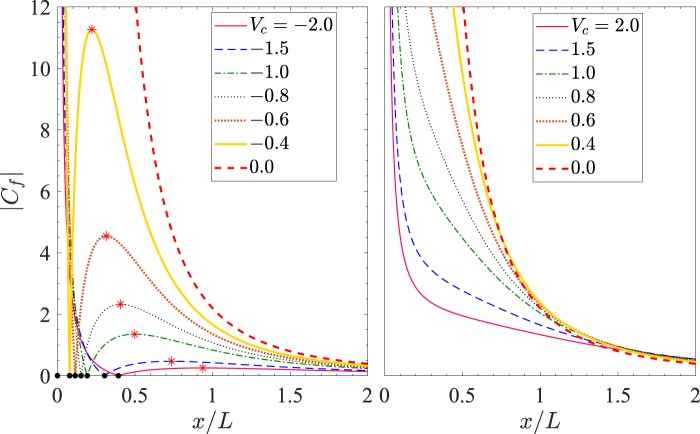
|*C*_*f*_| vs *x*/*L* for $$ {\mathcal M} =1$$, $$ {\mathcal R} e=2$$, and *n* = 1.5.

Beyond *x*/*L* = *x*_*m*_/*L*, |*C*_*f*_| falls down with further increase in *x*/*L* and approaches the value 0 at about *x*/*L* = 1. The entire pattern of these curves shifts towards right in (*x*/*L*,*C*_*f*_)-plane on increasing *n*. Thus the magnitude of skin friction |*C*_*f*_| may increase or decrease on increasing *n*, depending upon the horizontal distance from the slit. Similar is the variation of *C*_*f*_ with *x*/*L* for the other negative values of *V*_*c*_. The dependence of |*C*_*f*_| on suction can be easily understood from Fig. 6, which has been drawn for *n* = 1.5 and the fixed parametric values of ${\mathcal M}$ and ${\mathcal R} e$ same as in Fig. 5. Here in Fig. 6, each curve corresponds to one value of *V*_*c*_. It is clear from the first subfigure of Fig. 6 that on increasing suction, the point of maximum (*x*_*m*_,|*C*_*f*_(*x*_*m*_)|) shifts downward in the (*x*/*L*,*C*_*f*_) plane and for any fixed value of *x*/*L* sufficiently away from the slit, |*C*_*f*_| decreases on increasing suction. This is due to the comparative values of the two terms −*f* ′′(0) and (*n*−1)*V*_*c*_/(*x*/*L*)^(*n*+1)/2^ in shear stress *τ* or in *C*_*f*_ as can be seen from (41).

We now get back to Fig. 5 to explain the case of injection (*V*_*c*_ > 0) for which the behavior of *C*_*f*_ is different from the case of suction. Here, *C*_*f*_ remains positive for all *x* > 0. For any fixed value of *n*  ≥  1, the skin friction coefficient *C*_*f*_ is a decreasing function of *x*/*L* in the considered range of *x*/*L* and *C*_*f*_. By correlating Figs. 5 and 6 it can be observed that the skin friction decreases on increasing injection. This observation is also in accordance with the physical expectation that the injection at the sheet wall enhances the vertical component of the fluid velocity near it, which in turn results in lowering of the frictional drag near the wall.

**Figure 7:**
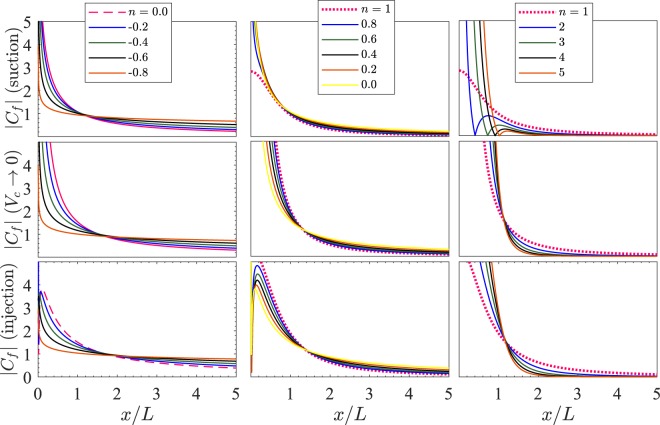
|*C*_*f*_| vs *x*/*L* for $$ {\mathcal M} =1$$, $$ {\mathcal R} e=2$$. For suction *V*_*c*_ = −1 and for injection *V*_*c*_ = 1.

**Case II:** 0 ≤ *n* < 1. Observe from (41) that for (*n*−1) < 0, the term (*n*−1)*V*_*c*_ is positive for suction while it is negative for injection. Here, the effect of change of *n* is more prominent in the case of injection than that in suction in view of the variation of |*C*_*f*_| with *x*/*L* (see the second column of Fig. 7).

**Case III:** −1 < *n* < 0. On referring back to (41) once more, one can check that here, for a fixed value of *n* between −1 and 0, a substantial variation of |*C*_*f*_| with *x*/*L* can be seen. This variation is comparatively lesser in the case 0 ≤ *n* < 1 as can be observed by comparing the first and second columns of Fig. 7. For any fixed value of *V*_*c*_ and *x*/*L*, the magnitude of |*C*_*f*_| decreases with *x*/*L* near the slit and increases for a distance sufficiently away from the slit as *n* is varied from 0 to *n* → −1. Note from (33) that

44 $$\mathop{{\rm{l}}{\rm{i}}{\rm{m}}}\limits_{n\to -{1}^{+}}\{-\sqrt{n+1}{f}^{{\rm{^{\prime} }}{\rm{^{\prime} }}}(0)\}\approx \sqrt{\frac{2(3 {\mathcal M} -2)}{3}},\, {\mathcal M}  > 2/3$$

which is independent of suction and injection. Also note that for ${\mathcal M}  > 2/3$, |*f* ′′(0)| rises rapidly (hence *x*_0_ falls rapidly) as *n* is varied from 0 to −1. Using (44) in (41), we have the following

45 $$\mathop{{\rm{l}}{\rm{i}}{\rm{m}}}\limits_{n\to -{1}^{+}}{C}_{f}\approx 2\sqrt{\frac{3 {\mathcal M} -2}{3{\mathscr{R}}e}},\, {\mathcal M}  > 2/3.$$

From (45), we have for ${\mathcal M} =1$ and ${\mathcal R} e=2$, ${C}_{f}\to \sqrt{\frac{2}{3}}\approx 0.81649659$ as *n* → −1^+^ for all values of *V*_*c*_ and all *x* > 0. On comparing all the three columns of Fig. 7, it can be inferred that the unusual behavior of significant increase or decrease of |*C*_*f*_| with *x*/*L* is apparent in the case of (i) suction for *n* > 1 (ii) injection for 0 < *n* < 1 and (iii) both suction and injection for *n* < 0. These observation are useful to model the nonlinearity of the underlying boundary layer flow for the two cases of suction and injection using an appropriate value of the stretching sheet parameter *n*.

### Effect of Reynolds number

**Figure 8:**
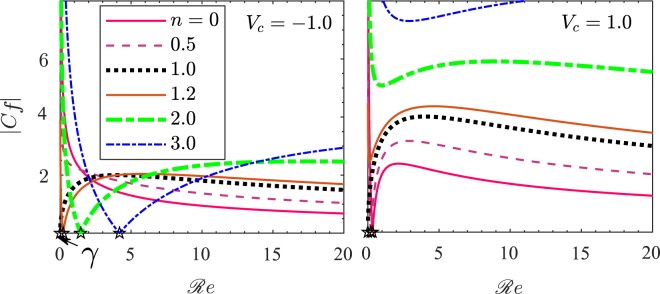
*C*_*f*_ vs ${\mathcal R} e$ for different values of *n*. The fixed parametric values are ${\mathcal M} =1$ and *x*/*L* = 0.5.

To see the effect of Reynolds number ${\mathcal R} e$ on the skin friction near the wall, we have obtained Fig. 8, which shows the variation of |*C*_*f*_| with ${\mathcal R} e$ for various values of *n* and *V*_*c*_. The fixed parametric values are ${\mathcal M} =1$ and *x*/*L* = 0.5. For a fixed value of *V*_*c*_, the different curves correspond to different values of *n* = 0, 0.5, 1, 1.2, 2, and 3. Observe from the expression for *C*_*f*_ in (41) that for a given value of *x*/*L*, *C*_*f*_ = 0 for ${\mathcal R} e=\gamma$, where

46 $$\gamma =\frac{(n-1){V}_{c}}{-2f^{\prime\prime} (0)}{(x/L)}^{-(n+1)/2}.$$

For ${\mathcal R} e\in \mathrm{(0,}\gamma )$, *C*_*f*_ < 0, whereas *C*_*f*_ > 0 for ${\mathcal R} e > \gamma$. However, for *V*_*c*_ = −1, *C*_*f*_ remains positive for all permissible values of ${\mathcal R} e$. For a fixed value of *n* in the range 0 ≤ *n* ≤ 1, |*C*_*f*_| decreases continuously with ${\mathcal R} e$ in the case of suction (*V*_*c*_ = −1), while in the case of injection (*V*_*c*_ = 1), *C*_*f*_ increases with ${\mathcal R} e$ initially, attains rapidly a maximum and then it decreases slowly with ${\mathcal R} e$. For *n* > 1 and *V*_*c*_ = −1, |*C*_*f*_| decreases rapidly with ${\mathcal R} e$ to 0 and then starts increasing with ${\mathcal R} e$ and slowly attains a maximum value at larger Reynolds number, after which it decreases further on increasing ${\mathcal R} e$. The variation for the case of injection is similar as it is for *n* < 1 except that on increasing *n*, |*C*_*f*_| increases for a fixed value of ${\mathcal R} e$.

### Role of magnetic parameter ${\mathcal M}$

**Figure 9:**
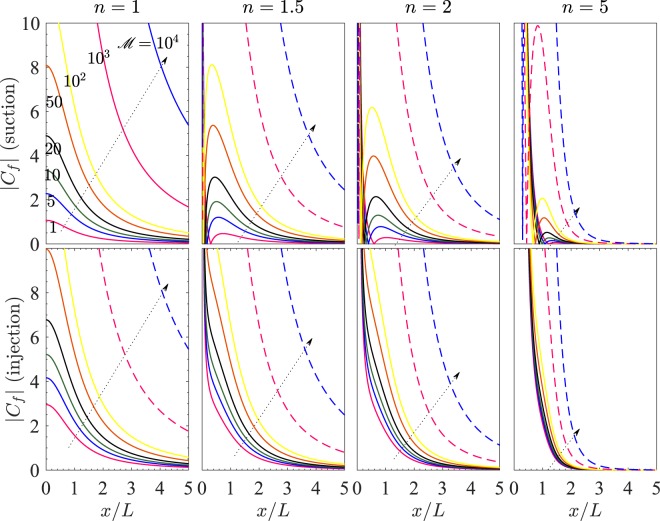
*C*_*f*_ vs *x*/*L* for different values of ${\mathcal M}$ and *n*. Here, *V*_*c*_ = −1.5 for suction and *V*_*c*_ = 1.5 for injection.

The effect of ${\mathcal M}$ on *C*_*f*_ can be observed from (33). Clearly, |*f*′′(0)| is an increasing function of ${\mathcal M}$. Larger ${\mathcal M}$ contributes towards greater resistance to the flow in *x*-direction. This indicates that the skin friction near the sheet wall is expected to rise for *x*/*L* away from 0 when ${\mathcal M}$ is sufficiently large. To see this effect explicitly, we have obtained Fig. 9 which shows variation of *C*_*f*_ with *x*/*L* for ${\mathcal R} e=2$, where we have taken *V*_*c*_ = −1.5 for suction and *V*_*c*_ = 1.5 for injection. In each of the case for suction/injection, the subfigures have been drawn for *n* = 1, 1.5, 2, and 5. In each of the subfigures, the different curves correspond to ${\mathcal M} =1$, 5, 10, 20, 50, 10^2^, 10^3^, and 10^4^. Each arrow head denotes the direction of increase of the magnetic parameter ${\mathcal M}$.

For the case of suction, for *n* = 1 and for each value of ${\mathcal M}$, |*C*_*f*_| is a decreasing function of *x*/*L*. However, the variation of |*C*_*f*_| with *x*/*L* is different for *n* > 1, where |*C*_*f*_| decreases on increasing *x*/*L*. The variation of |*C*_*f*_| for *n* = 1.5, 2, and 5 is similar, but the maximum of |*C*_*f*_| decreases on increasing *n*.

For the case of injection, *C*_*f*_ remains positive for all the considered values of *n* and ${\mathcal M}$. In either of the cases of suction and injection, we see that |*C*_*f*_| increases significantly on increasing ${\mathcal M}$, which shows enhancement in the skin friction caused by increasing the applied magnetic field and decreasing the permeability of the porous medium.

### Streamline pattern

**Figure 10:**
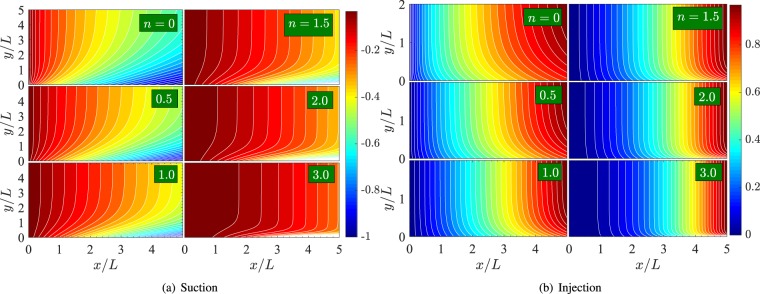
Streamline pattern in (*x*/*L*,*y*/*L*)-plane for (**a**) *V*_*c*_ = −1.5 and (**b**) *V*_*c*_ = 1.5. The fixed parametric values are, ${\mathcal R} e=2$ and ${\mathcal M} =2$.

Figure 10 shows the streamline pattern in the (*x*/*L*,*y*/*L*)-plane for (a) suction (*V*_*c*_ = −1.5) and (b) injection (*V*_*c*_ = 1.5) with the fixed parametric values of ${\mathcal R} e=2$ and ${\mathcal M} =2$. Each subfigure corresponds to one value of *n* among 0, 0.5, 1, 1.5, 2.0, and 3.0. We define the boundary layer thickness to be *h*(*x*), such that as *y* → *h*(*x*), $\frac{\partial u}{\partial y}\to 0$ and for *y* > *h*(*x*), *u* = 0. So in the region *y* > *h*(*x*), the stream lines will be vertical. In the presence of suction at the boundary, the fluid particles in contact with the sheet start moving with the velocity of the sheet, advance towards left-upward before they are along the path normal to the sheet surface. The particles farther from the slit move through greater distance along the inclined curves. So the boundary layer thickness *h*(*x*) increases with the distance from the slit. This happens for all the considered values of *n* and is due to the strengthened fluid sheet velocity. These observations together show that the boundary layer flow in the presence of suction is significantly affected by changing the stretching parameter *n*.

On the other hand, in the presence of injection at the sheet (Fig. 10(b)), the fluid particles in contact with the sheet start moving with the velocity of the sheet, advance a little towards right-upward and attain paths normal to the sheet. Here, the boundary layer thickness *h*(*x*) is smaller than in the case of suction, where at a fixed distance from the slit, *h*(*x*) diminishes with increase in *n*. For each value of *n*, *h*(*x*) increases with the distance from the slit.

**Figure 11:**
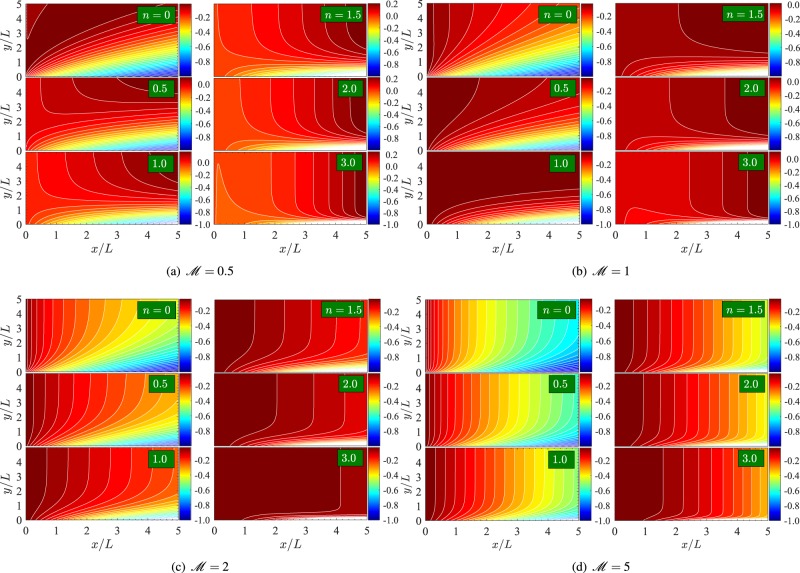
Streamline pattern in (*x*/*L*,*y*/*L*)-plane for *V*_*c*_ = −1 and ${\mathcal R} e=2$.

To observe the effect of porosity and applied magnetic field on the boundary layer flow pattern, we have obtained Fig. 11 which shows the streamline pattern in the *xy*-plane, for different values of *n*, *V*_*c*_ = −1 and ${\mathcal R} e=2$. The various patterns (a)–(d) correspond to ${\mathcal M} =0.5$, 1, 2, and 5 respectively. Clearly, for a fixed value of *n*, the boundary layer thickness decreases on incrementing ${\mathcal M}$ from 0.5 to 5.

**Figure 12:**
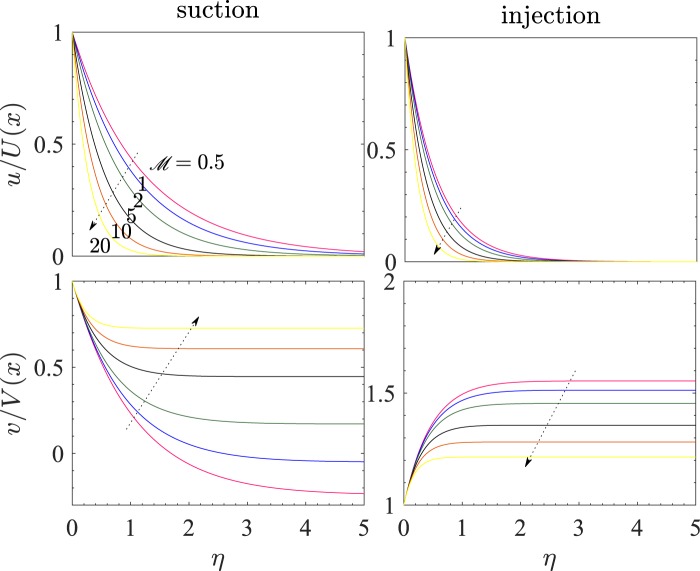
Velocity profiles *u*/*U*(*x*) and *v*/*V*(*x*) vs *η* for suction (*V*_*c*_ = −1) and injection (*V*_*c*_ = 1) and ${\mathcal R} e=2$.

It will be useful to see the explicit dependence of the velocity profiles on ${\mathcal M}$ for the two cases of suction and injection. This has been shown in Fig. 12, which shows the variation of the two components of velocity, *u*/*U*(*x*) and *v*/*V*(*x*) with *η* for *n* = 1.5 and ${\mathcal R} e=2$. Here, we have taken *V*_*c*_ = −1 for suction and *V*_*c*_ = 1 for injection. Each curve is drawn for one value of ${\mathcal M}$ among 0.5, 1, 2, 5, 10, and 20, and the arrowhead in each subfigure shows the direction of increase of ${\mathcal M}$. Clearly, on increasing ${\mathcal M}$, the horizontal velocity profile shifts downwards in the (*η*,*u*/*U*(*x*))-plane under suction as well as injection. On the other hand, the profile for the velocity component *v*/*V*(*x*) goes up on increasing ${\mathcal M}$ for the case of suction, while opposite to this variation occurs under injection. On correlating all the four subfigures, we see that under suction, the applied magnetic field and the permeability of the porous medium tend to enhance the fluid velocity in the direction of suction, while it hinders the fluid motion along the sheet. Under the case of injection, the applied magnetic field hinders both the vertical as well as horizontal movement of the fluid in the vicinity of the sheet wall. The value of *y* corresponding to least value *η* for which *u*/*U*(*x*) becomes zero, is the boundary layer thickness. So increase in ${\mathcal M}$ decreases the boundary layer thickness.

## Conclusions

The present work generalizes the classical work of Crane^7^, Pavlov^9^, Gupta and Gupta^34^, Hayat *et al*.^14^, and Rashidi^15^ on the flow of Newtonian fluid driven by stretching sheet with external magnetic field through porous medium with suction/injection. The underlying dynamical system is described by the nonlinear boundary layer equations, which are transformed into a system of nonlinear ordinary differential equations via similarity transformations. The resulting system is solved analytically and numerically using highly efficient HAM.

The second order analytical solution suggests that the axial and transverse velocities of the boundary layer flow can be approximated by a finite linear combination of the basis functions {exp{−*kη*}}_*k* ≥ 0_, where the coefficients are functions of the dimensionless parameters *n*, *V*_*c*_, ${\mathcal R} e$, and ${\mathcal M}$. The method allows a very good approximation of the second derivative *f* ′′(0) through the formula (33). The numerical results have been obtained for a wide range of parametric values.

An increase in the values of the ${\mathcal M}$ results in pulling down of the horizontal velocity profiles of the flow. This may be due to an increase in the Lorentz and Darcy forces in the fluid, which oppose the fluid motion. An increase in the parameter ${\mathcal M}$ leads to rise in the skin friction in all the cases of suction and injection.

An increase in the numerical values of *V*_*c*_ and ${\mathcal M}$ results in enhancement in the horizontal component of the fluid velocity due to which the laminar MHD boundary layer gets depleted.

The present numerical results are useful in modeling the nonlinearity of the underlying boundary layer flow for the two cases of suction and injection using an appropriate value of the stretching sheet parameter *n*. More precisely, it may be useful to take *n* > 1 for the case of suction, 0 < *n* < 1 for injection, and −1 < *n* < 0 both for suction and injection in order to observe the modifications occurring in the boundary layer flow.

The asymptotic behavior of the solution corresponding to the boundary layer flow near the stretching sheet surface in the presence of suction/injection is discussed separately for (i) ${\mathcal M}$ large and (ii) *V*_*c*_ large. The approximated analytic solution is found to be in excellent good agreement with the numerical solution obtained without any approximation for ${\mathcal M}$ of the order of 10^2^ and *V*_*c*_ of the order of 10^1^.

Since the linear stretching can be acknowledged in practice only with great care and meticulous effort, the consideration of the nonlinear stretching sheet is more useful.

## Footnotes

1 In the present work, we have used the homotopy perturbation method which is a particular case of the more general homotopy analysis method (HAM) developed by Prof. Liao^24^. So we prefer to use the phrase HAM throughout.
